# Reproductive biology and anatomy of ammonites

**DOI:** 10.1038/s41598-025-23299-y

**Published:** 2025-11-12

**Authors:** Christian Klug, Günter Schweigert, René Lauer, Bruce Lauer, Dirk Fuchs, Kan Terakado, Amane Tajika

**Affiliations:** 1 Paläontologisches Institut und Museum, Universität Zürich, Karl-Schmid-Strasse 4, 8006 Zürich, Switzerland; 2 Staatliches Museum für Naturkunde, Rosenstein 1, 70191 Stuttgart, Germany; 3 Lauer Foundation for Paleontology, Science and Education, Wheaton, IL 60189 USA; 4 SNSB-Bayerische Staatssammlung für Paläontologie und Geologie, Richard-Wagner-Straße 10, 80333 Munich, Germany; 5 Graduate School of Human and Environmental Studies, Kyoto University, Yoshida Nihonmatsu-Cho, Sakyo-Ku, Kyoto, 606-8316 Japan; 6 Hakubi Center for Advanced Research, Kyoto University, Yoshida-Honmachi, Sakyo-Ku, Kyoto, 606-8501 Japan; 7 Division of Paleontology (Invertebrates), American Museum of Natural History, Central Park West 79Th Street, New York, NY 10024 USA

**Keywords:** Cephalopoda, Ammonoidea, Tithonian, Conservation deposits, Dimorphism, Anatomy, Ovaries, Palaeontology, Palaeoecology

## Abstract

Ammonoid anatomy is still poorly known and every new record of a specimen with soft tissue-preservation yields valuable information. In view of the impressive morphological disparity of ammonoids, we can also expect some disparity in soft tissue anatomy. Here, we present a new specimen from the Kimmeridgian of the Solnhofen region. In contrast to a recently described isolated soft body preserving the male reproductive organs, the new specimen for the first time shows a structure, which we interpret as the ovaries of the female containing immature eggs and further organs. These two specimens are of great importance for sexing ammonoids and for estimating fecundity. The high reproductive rate of Jurassic ammonites underlines their great abundance and the important role of juvenile ammonoids at the base of Devonian to Cretaceous food webs.

## Introduction

Ammonoids are among the most important fossil groups, considering their 340 million years of evolutionary history, great diversity, morphological disparity and key role in biostratigraphy. While the conchs and, to a lesser extent, sclerotized jaws of ammonoids are well documented, the soft-part anatomy is still known from a few tens of specimens only. These specimens usually exhibit phosphatized, carbonized or pyritized remains of internal organs that lack detailed anatomical features and are thus hard to interpret^1–13^. Notably, these organ remains are largely confined to regions at or posterior to the buccal mass. Contrastingly, reports of arms crown remains are limited to very faint remains^14^ and arm hooks of scaphitids^15^. Thus far, remains of the buccal mass and radula^14,16–25^, the digestive tract including oesophagus, crop, stomach and gut^14,20,23,26–31^, the gills^26,32,33^, muscle remains^7,27,34–36^, questionable eye capsules^14,26^, and reproductive parts^26,37–39^ were described. While the overall internal anatomy is not expected to differ profoundly from the cephalopod bauplan, the morphological peculiarities of many organs have a huge potential for ecological and evolutionary implications. For example, the presence of only two gills supports the inclusion of the Ammonoida in the Dibranchiata/Coleoidea^26,33,40^, which in turn exemplifies the significance of the number of arms. Based on an extant phylogenetic bracket, Klug & Lehmann^4^ had already suggested ten arms as the likely condition among ammonoids. *Gordoniconus*, one of the earliest coleoids known with soft parts^3^ displays arm remains suggesting ten arms as well. Stomach contents inform about diets (list in^4^) and reproductive organs help sexing specimens^41^ and assessing fecundity^42,43^.

The primary challenge with mollusc soft tissue-preservation is that often, although soft tissue remains may be preserved, especially in conservation lagerstätten, they are often invisible because they are covered by the shell. Most of the published internal organs of ammonoids are visible because the shell broke off (e.g^42^.,), the aragonitic shell is dissolved (e.g^14,19,27,31^.,), the specimens were sectioned (e.g^23,24,37,39^.,), synchrotron-scanned^15,21,34^ or the soft parts fell out of the shell prior to burial (‘pabulite’ sensu^26,41,44^).

Here, we present a specimen of the macroconchiate ammonite *Neocheotoceras* cf. *praecursor*^12^. The genus is commonly found in the platy limestones of the Solnhofen-Eichstätt region in southwestern Germany^45^. Its antidimorphic pairs (*Neochetoceras* spp. – *Lingulaticeras* ex gr. *solenoides*) are moderately well-documented^46,47^, although in the case of the specimen presented here, the corresponding microconchiate taxon is unclear. Further, it is one of the genera with the most widely documented stomach contents^48,49^,Lehmann 1976^9,50^;. For the first time, an ammonite exhibits some anatomical detail of the posterior internal organs, helping with the homologisation.

**Fig. 1:**
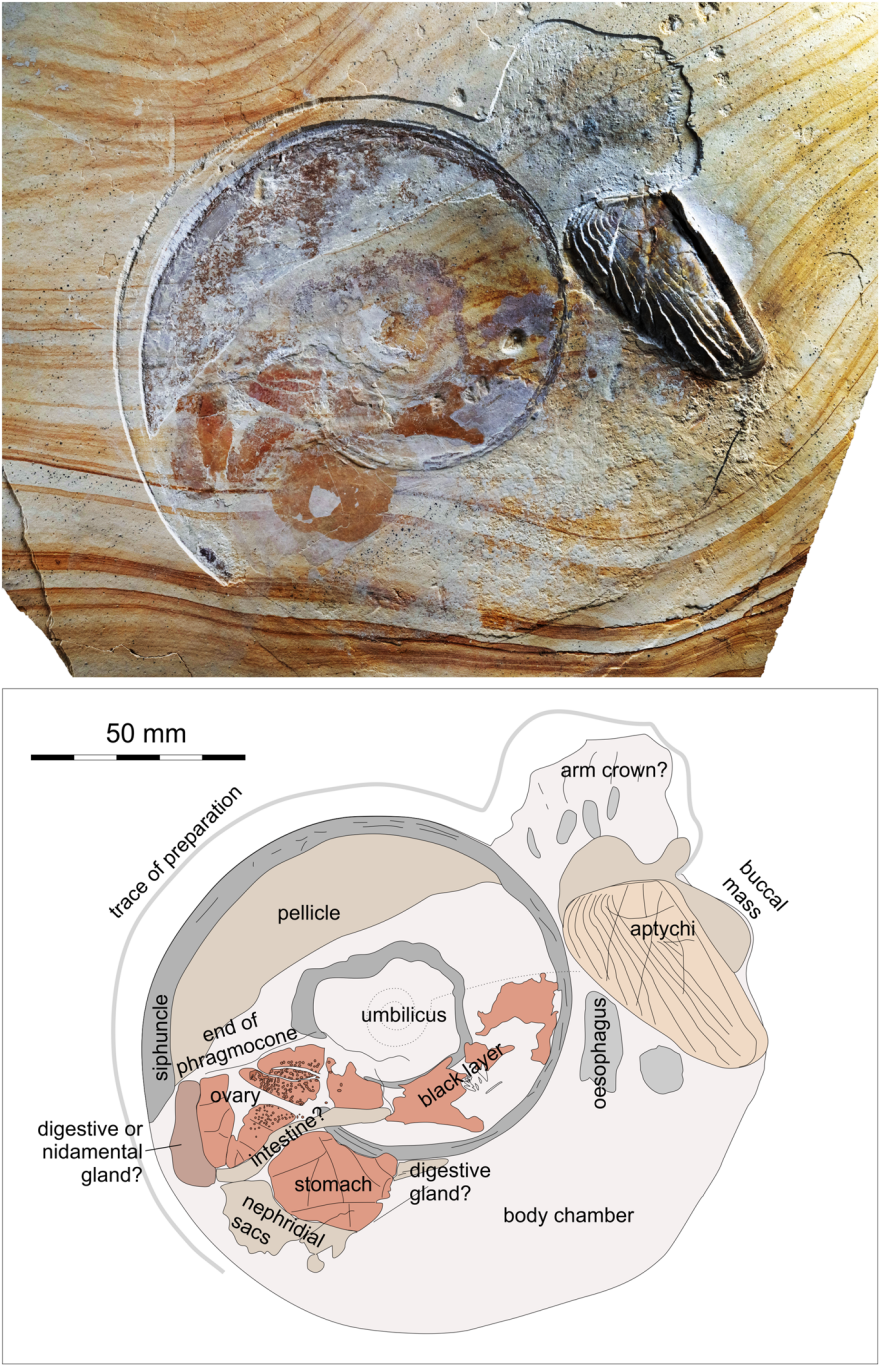
Flattened specimen of *Neochetoceras* cf. *praecursor*, Kimmeridgian, Öchselberg quarry, Germany. Top: The specimen was photographed under white light and the contrast was enhanced using PhotoShop CS2. Bottom: Drawing of the visible structures and a tentative homologisation of organs.

This new specimen (Fig. 1) originates from the Late Jurassic of Bavaria, Germany, as does the pabulite described by Klug et al^26^.. The specimen is important for several reasons: (1) it is preserved in situ with a conch imprint, (2) it exhibits potential arm crown traces, and (3) its internal organ remains are well-structured and show differences in preservation including remains of what might be ovaries with immature eggs. In addition to describing the fossil, we discuss (4) implications for ammonoid fecundity and dimorphism.

## Results

**Fig. 2:**
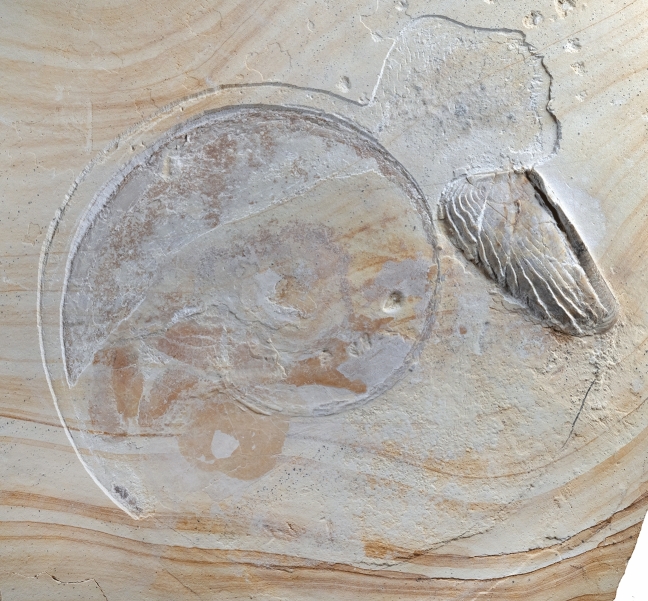
Flattened specimen of *Neochetoceras* cf. *praecursor*, Kimmeridgian, Öchselberg quarry, Germany. Top: The specimen was photographed under white light. Here, the colors and contrasts correspond to the specimen’s appearance.

**Fig. 3:**
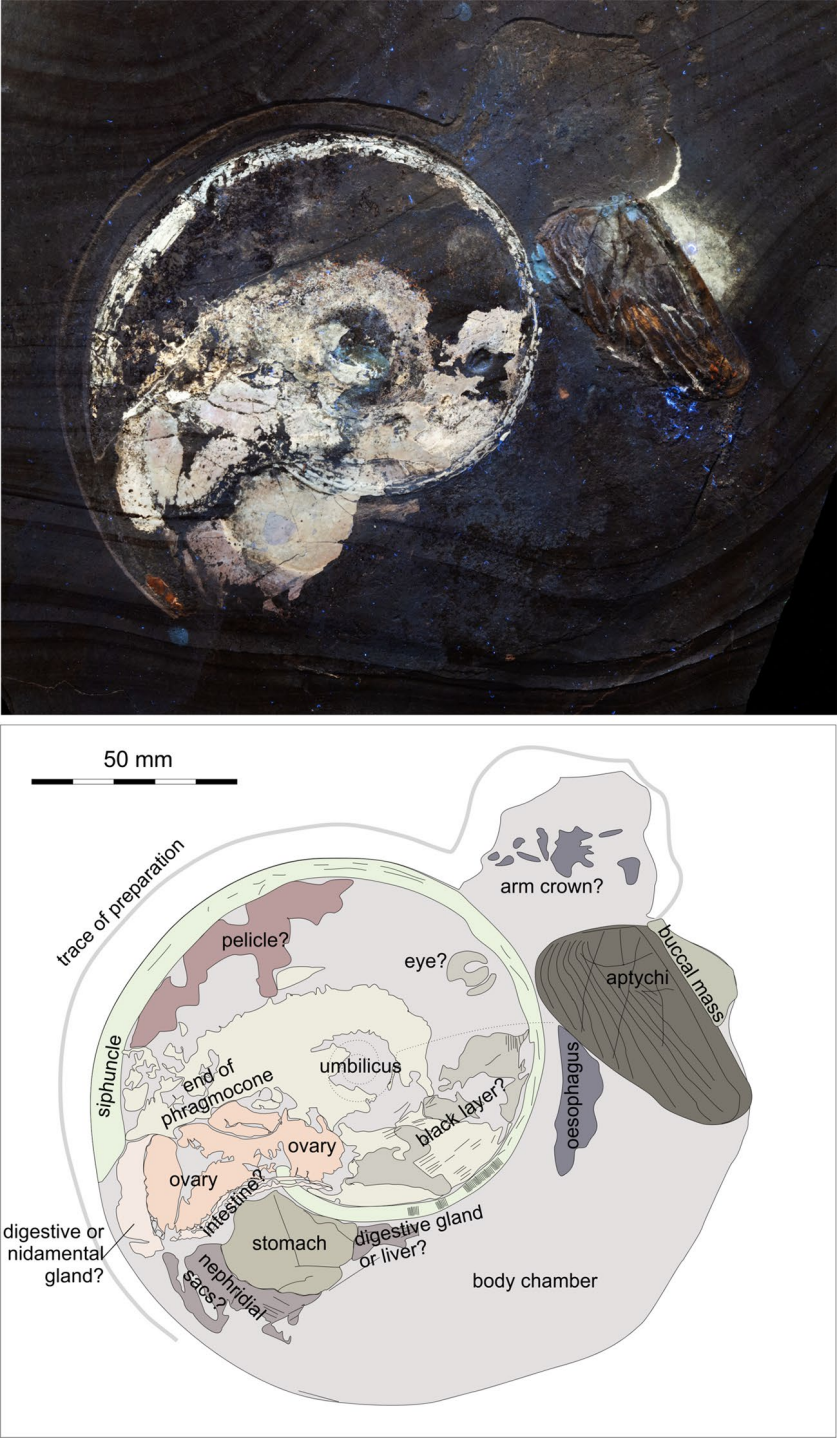
Flattened specimen of *Neochetoceras* cf. *praecursor*, Kimmeridgian, Öchselberg quarry, Germany. Top: The specimen was photographed under UV light and the contrast was enhanced using PhotoShop CS2. Bottom: Drawing of the visible structures and a tentative homologisation of organs.

The ammonite LF 1407 (Fig. 1, 2, 3) was partially covered by sediment and no counterslab is available. The conch outline measures about 120 mm in diameter. It is strongly flattened, and the coiling is mainly evident from the phosphatized siphuncle. The specimen preserves no conch ornament. The length of the calcitic aptychus is about 55 mm, which indicates the minimum whorl height. The corresponding upper jaw is not discernible in this specimen. Because of the combination of a narrow umbilicus and a smooth conch (compaction and shell dissolution may have removed fine ornament, though) with the *Lamellaptychus*, we assign this specimen to *Neochetoceras* cf. *praecursor*, a species recognized as macroconch by Scherzinger et al^47^.. The species of *Neochetoceras*usually have a narrow umbilicus, a moderately high whorl expansion of around 2.1 to 2.2 and a rather slender conch with a whorl height/diameter ratio below 0.2. Since the conch is flattened, we cannot determine these values directly and have to rely on published specimens (e.g^51^.,: Fig. 6A-C). Nevertheless, its overall conch shape is preserved well enough to allow this species assignment, also taking the stratigraphic and geographic origin into account.

The soft tissue preservation differs from that known from coleoid fossils of the Solnhofen-Eichstätt region^52,53^. This can be expected because the preservation of organs depends on their primary composition and the preservation of the whole animal depends largely on its physiology^54^. Accordingly, we think that ammonoid soft tissues preserve in different ways than those of coleoids or nautilids^44^.

More importantly, the specimen displays a series of phosphatized structures in the body chamber, mostly in the posterior quarter and in the whorl overlap zone. These fossilized tissues can be discerned both in the white light (Figs. 1, 2) and the UV-light photos (Fig. 3). Such phosphatized soft tissue remains can be found occasionally in ammonoids from platy limestones. However, these remains are usually hard to identify in the absence of anatomical details. In this specimen, the phosphatized structures vary in colour both under white and UV light. Additionally, some exhibit finer details, which we interpret as remains of primary organic structures. While some of the fossilized structures can be readily homologized with cephalopod organs, others lack sufficient anatomical detail for a definitive interpretation. In these cases, we discuss the homology criteria (position, specific quality, continuity) in detail (see also^26^). We subdivide and discuss the soft tissue remains according to their location.

### Phragmocone

1a. A ventral spiral structure with longitudinal cracks: This stripe of phosphate in ventral position can be identified confidently as siphuncle (for soft-tissue preservation of siphuncles see^11^ and^55^).

1b. A much broader, thickly mineralized band: we interpret this as the black band since it appears to be limited to the whorl overlap zone^56^.

1c. A dark brown structure in ventral position of the last demi-whorl of the phragmocone. Since this is far in front of the aperture, it cannot be the black band. In the greater Solnhofen region, ammonoids often carry a thin sheet of phosphate on their phragmocone. We suggest that these are remains of organic sheets occurring inside the chambers, the pellicle.

### Posterior body chamber

**Fig. 4:**
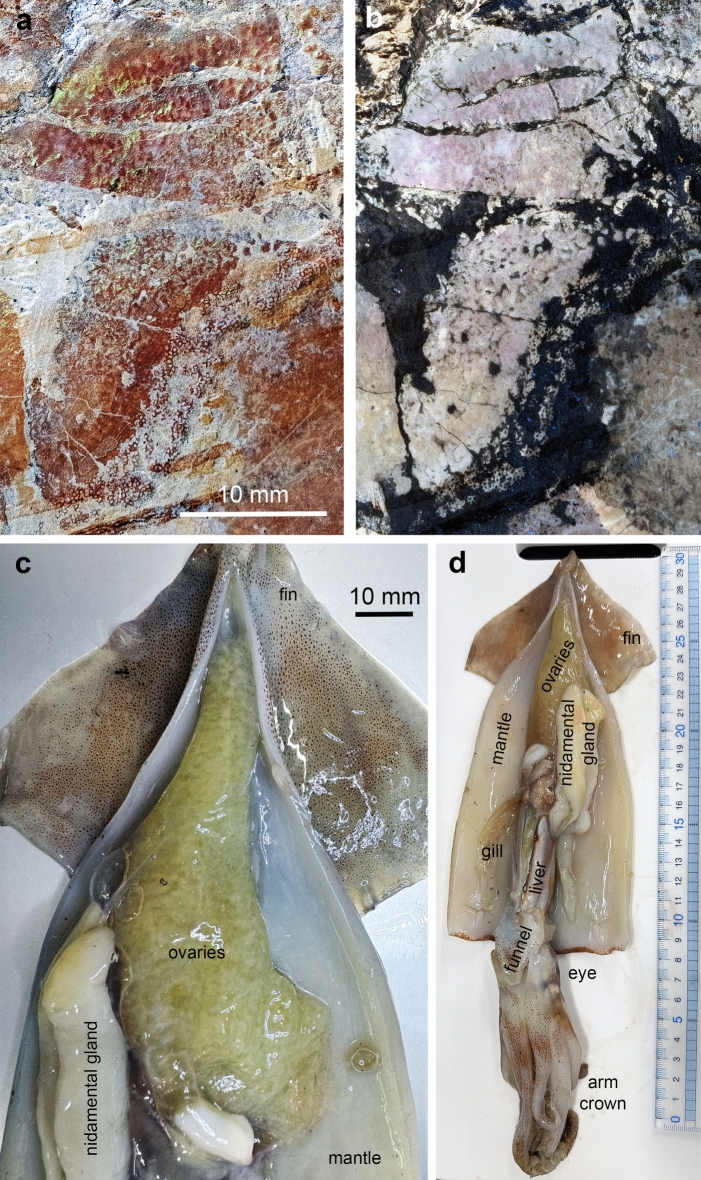
Comparative anatomy of the Jurassic ammonite *Neochetoceras* cf. *praecursor* and the Recent decabrachian *Todarodes pacificus*. a, b, ovary and? intestine of *N.* cf. *praecursor*. (**a**) white light. (**b**) UV-light. c, d, dissected specimen of *T. pacificus*. (**c**) detail of (**d**) showing the ovaries and the nidamental gland. d, the entire squid.

**Fig. 5:**
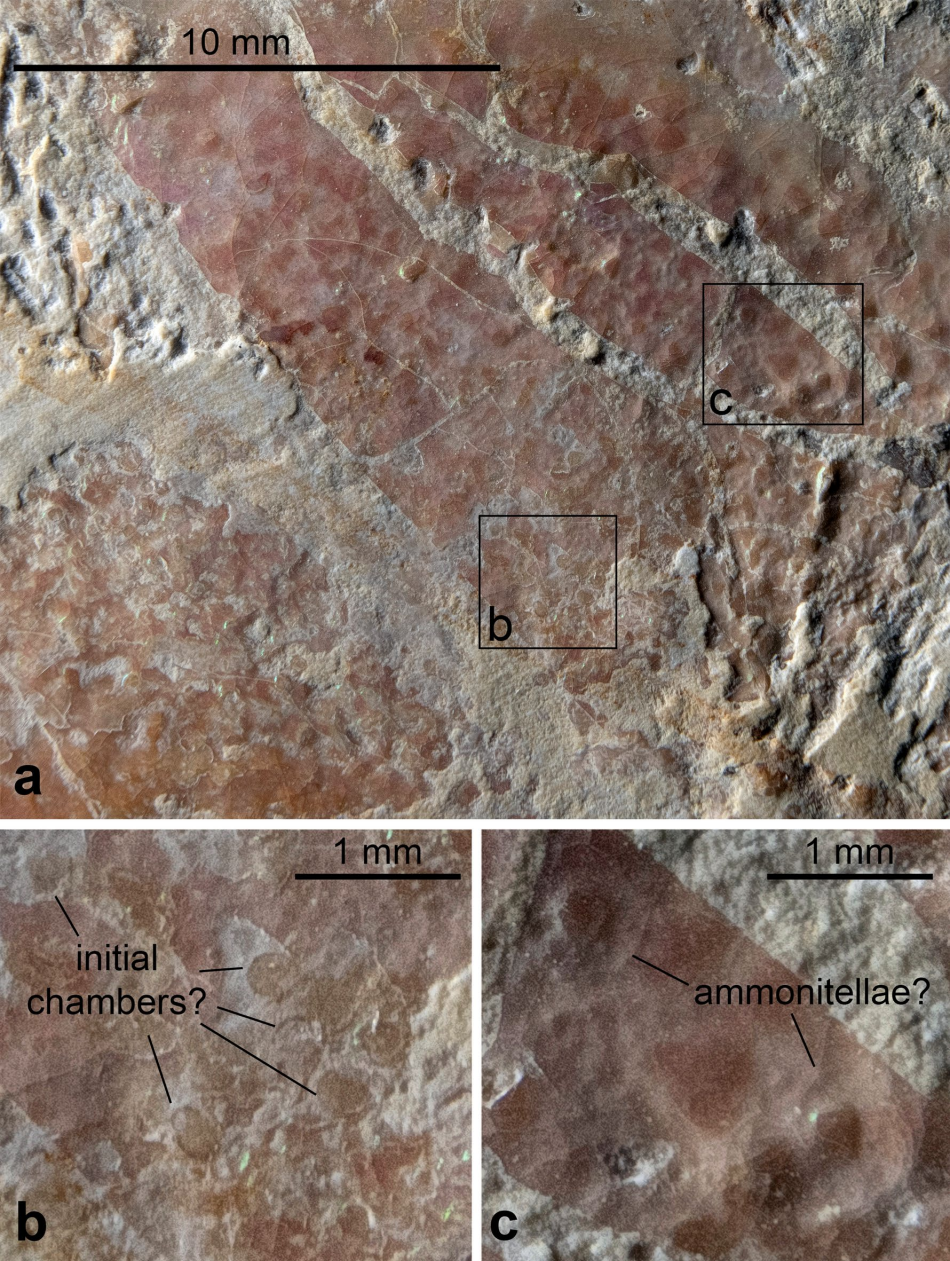
Details of the supposed ovaries of *Neochetoceras* cf. *praecursor*, Kimmeridgian, Öchselberg quarry, Germany. The specimen was photographed under white light. (**a**) part of the supposed ovaries; note the varying size of the round structures. (**b**) detail of **a** with subcircular to oval structures; note the dark edges. (**c**) detail of **a** with larger structures resembling ammonitellae with a darker center, possibly representing the initial chambers.

**Fig. 6:**
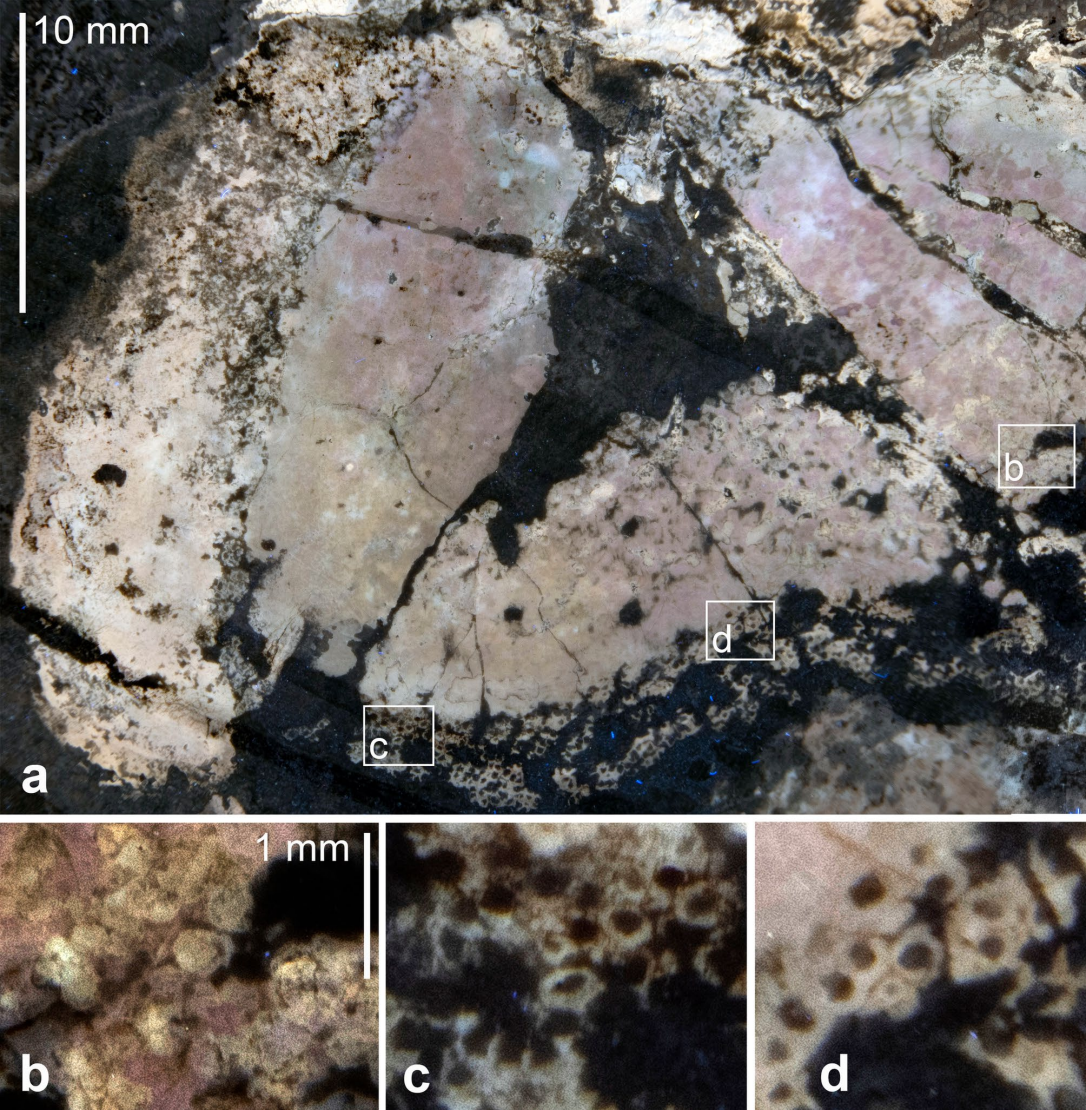
Details of the supposed ovaries of *Neochetoceras* cf. *praecursor*, Kimmeridgian, Öchselberg quarry, Germany. The specimen was photographed under UV light. (**a**) part of the supposed ovaries; note the varying size of the round structures. (**b**), (**c**), (**d**), details of **a** with subcircular to oval structures; note the distinct outer walls. The light grey colour under UV suggests that the walls in **c** and **d** are now phosphatized.

2a. A spotted series of brownish patches in posterior dorsolateral position. These structures cover a surface of about 50 × 25 mm. The darker spots within these patches measure 0.4 to 1 mm across and are better visible in the white light photo (Fig. 4a) and macro details (Figs. 5b, c, 6c-e). The surface displaying these spots is subdivided into four fields (probably by beginning decay), in which these patches differ in appearance. Three of these fields run subparallel to each other and can be seen in Fig. 4a in the upper half of the photo. In the upper two fields (upper right corner in Figs. 5a, 6a), the brownish patches are moderately regularly distributed with slightly brighter parts between.

The third field has a more blurred appearance but still shows the brownish patches, particularly in the anterior part (lower right in Figs. 5a, 6a). The posterior part displays a nearly polygonal pattern with fields, resembling those of the structure described in 2c in size. In the fourth field, some regions show similar brownish patches, while the anteroventral part has a blurred brown appearance with faint bright patches in it. These patches correspond in size to those of the structure described in 2c.

In many modern molluscs like gastropods, bivalves or cephalopods, the ovaries are situated quite deeply within the body, where they are best protected. This is also true for modern nautilids^57^ and coleoids^58^. However, in nautilids, the ovaries lie below the digestive glands^57^, while in coleoids, they are in a dorsal position^58^. Since ammonoids share the ancestors with coleoids^59^, we postulate a posterodorsal position of the ovaries. Hence the homology criterion of position is fulfilled. Concerning the specific qualities, the size corresponds well to the ovaries of, e.g. modern coleoids and nautilids. KT and AT dissected a female *Todarodes pacificus* (Fig. 4c, d) to document the ovaries as a modern example for coleoids^60^. The immature eggs create a pattern, which is quite similar to that of the ammonite organ in shape and proportions. An independent test was carried out by presuming that this is a macroconch specimen. Scherzinger et al. (^47^: Fig. 8) illustrated the dimorphic couple and enabled us to identify the specimen as macroconch, which supports our interpretation. Accordingly, we conclude that these are the ovaries.

The round patches within vary in size between c. 0.25 and 1 mm (Figs. 5b, c, 6b, c, d). According to De Baets et al. (2015), the initial chamber of Jurassic ammonoids measures between 0.2 and 0.8 mm, while the whole ammonitella is usually between 0.3 and 1 mm in diameter. This suggests that the patches correspond to different developmental stages from such with mainly the initial chamber (Figs. 5b, 6b, c, d) to such where more or less the complete ammonitella was already present (Fig. 5c). 2b. A ventral patch of dark brownish colour (c. 25 × 10 mm), which fades out posteriorly: This patch lacks finer structures, which hampers its homologisation. In modern nautilids, the pericardial appendages and digestive glands can be found in a similar position^57^: Fig. 1). In coleoids, however, the nidamental glands are located anteroventrally of the ovaries. Of course, in a conch with a compressed whorl cross section profoundly different to that of nautilids and the roughly conical mantle of squids, we have to expect some anatomical differences in organ arrangement and shape. Thus, we tentatively suggest that it is part of the digestive glands or the nidamental glands but with great reservation.

2c. A c. 5 mm wide and almost 50 mm long band crosses other structures from ventrolateral towards near the umbilical wall in an arc. It is characterized by numerous, quite regularly arranged brown circles of about 0.25 to 0.5 mm diameter: When comparing this structure to the soft tissue-ammonite described by Klug et al^26^., its position and proportions resemble those of the intestine. In modern nautilids, the distal intestine carries many parallel folds^57^: Fig. 8D), which are distinct from the small circular structures seen in the fossil. A remotely similar structure of small pits occurs in the spadix of modern nautilids^57^: Fig. 13G). However, in the spadix, these pits are arranged in regular rows. Also, this would contradict the ovary interpretation and notion that the macroconchs were the females. We propose two plausible interpretations: Either it represents the intestine with a differently folded inner or outer surface or food particles that created this pattern, or it is still part of the ovaries containing the smallest still immature eggs.

2d. Another brownish patch of rounded subrectangular outline (c. 20 × 30 mm) directly anterior to the structure described in 2c. Its surface hardly shows any structures. Its peculiar colour is reminiscent of the structure interpreted as ammonite stomach by Klug et al^26^.. Further, it corresponds in position reasonably well to stomachs with content (Fig. 4) that have been described from ammonites of the Solnhofen region^4,61^. Remarkably, *Neochetoceras* is the ammonite genus that has been the most often published with preserved stomach content^9,49,50,62^. Hence, we favour this interpretation.

2e. A very lightly greyish surface (mauve under UV-light, 35 × 15 mm) ventral of the structure described in 2c. Its ventral position corresponds somewhat to that of the nephridial sacs (= coelomic cavities) in squids, but the fossilized organ lacks further detail to provide support for this interpretation.

2f. A structure (c. 5 × 10 mm) similar in colour to that described in 2e, but anterior to the structure described in 2d. Either it is part of the same organ as in 2e or something else. Anterior to the stomach and below the oesophagus, the liver and the digestive gland is situated in modern squids such as *Todarodes*.

### Structures around the aptychi (head region)

3a. White band (10 × 20 mm) on the midflank posterior to the aptychi (only visible under white light). Its direct association with the buccal mass and elongate shape suggests that this could well be a part of the oesophagus. Due to its chitinous lining, it has a rather high preservation potential^26^.

3b. A bright patch anteroventral of the aptychi (c. 10 × 20 mm), visible both under UV and white light. Anatomically, two organs can be expected in this position: remains of the buccal mass or the hyponome. Since the shape is rather rounded than tubular or rectangular, we interpret it as part of the buccal musculature.

**Fig. 7:**
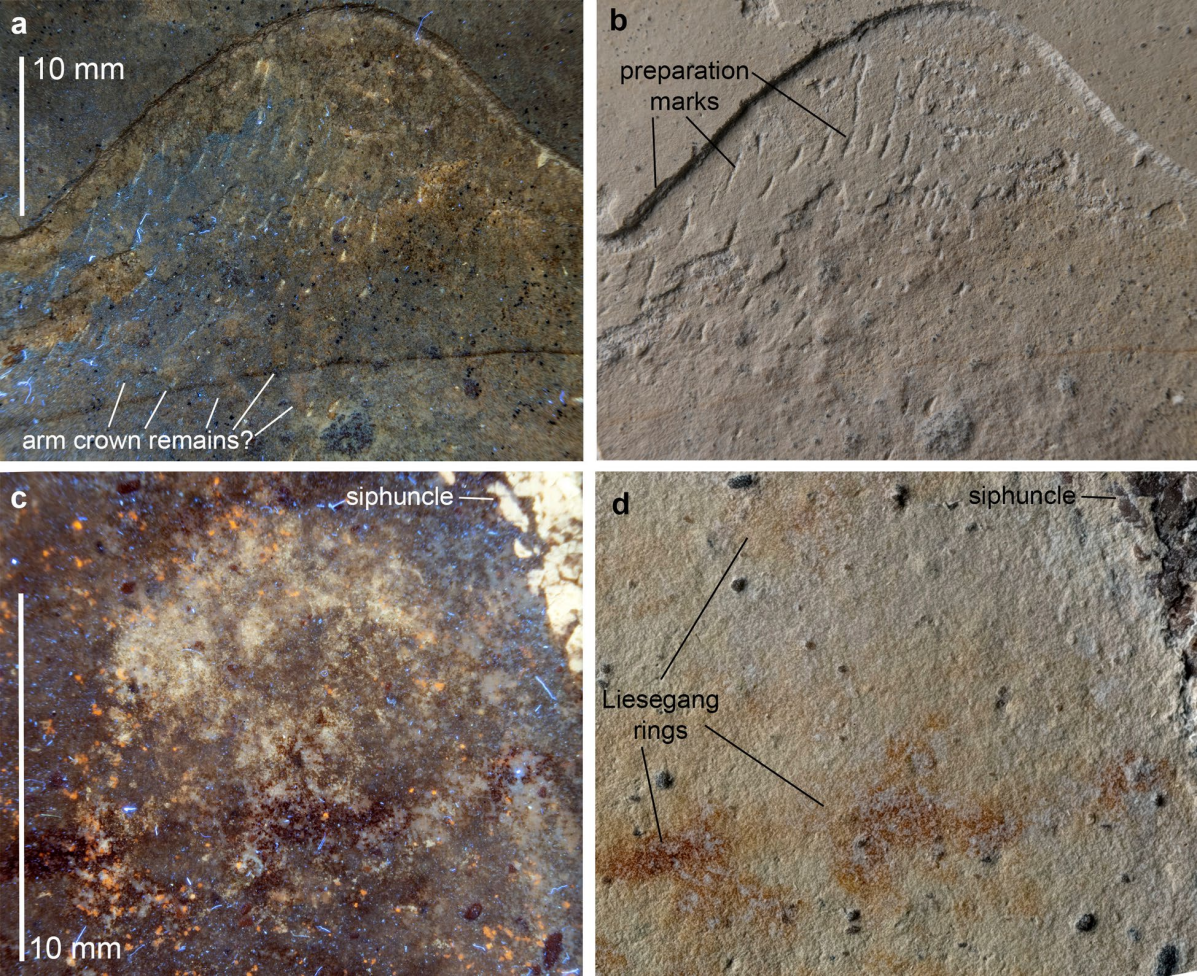
Flattened specimen of *Neochetoceras* cf. *praecursor*, Kimmeridgian, Öchselberg quarry, Germany. The specimen was photographed under UV light (**a**, **c**) and white light (**b**, **d**). In **c**, the contrast was slightly enhanced using PhotoShop CS2. **a, b**, surface anterior to the aptychi with potential arm crown remains. In addition to little calcite concretions, there are faint brownish patches, but their nature is uncertain in the absence of morphological detail. **c, d**, structure located dorsally of the aptychi on the phragmocone; the subcircular structure with a subcircular shade in its center maybe eye remains, which coincides with the ocular sinus in the aperture.

3c. A series of concretion-like elevations up to 10 mm long and a few millimetres wide anterior to the aptychi (Fig. 7a, b). Faintly visible under the different light sources. In the absence of clear outlines and internal structures, the interpretation depends on the position and rough structures. Anterior to the buccal mass, there is usually only the arm crown, which represents our best guess in this case. Unfortunately, the preservation is so poor that no further insights into the anatomy of this organ in ammonoids can be obtained. Nonetheless, these remains imply that the arms were somewhat short and had a low number, which would align with the extant phylogenetic bracketing of Klug et al. (2015) suggesting they had ten arms.

3d. Dorsal of the anterodorsal corner of the aptychi, there is a faint subcircular patch of about 12 mm diameter with a darker circle in the centre, moderately distinct under UV-light and very faint under white light (Fig. 7c, d). Its subcircular outline, position dorsolateral to the buccal mass and its appearance resemble the eyes fossilized in nautilids from the Cenomanian of Lebanon^44^. If the ventrolateral sinus in the shell accommodated the hyponome, a dorsolateral position of the eyes appears likely.

#### Ammonoid ovaries

Structures interpreted as ovaries or other structures related to reproduction in female ammonoids inside the conch have been recorded only extremely rarely. Ovoviviparity was discussed based on a female ammonite with numerous embryos in the posterior body chamber of a *Sinzovia* from the Cretaceous of Russia^42^. The conchs of these embryonic ammonites preserve in situ-aptychi that were already mineralized. In the sixties’, supposed ovaries were reported twice^37,39^. The acid preparation of the body chamber of a Jurassic *Eleganticeras* revealed an 8 mm long and 6 mm wide structure with numerous spherical structures with a diameter of 0.5 mm^37^. Accordingly, the size of the supposed eggs coincides with those found in the specimen presented here. These potential ovary remains are preserved in three dimensions, which makes the direct comparison difficult. Importantly, these remains are proportionally much smaller than the structure interpreted here as ovaries of *Neochetoceras*. However, Lehmann stated that only a part of the organ was prepared and that it may have been much larger.

In the second specimen, from which a supposed egg sac was reported, the egg sac shape is remarkably similar in appearance. It was found in a Triassic *Ceratites* of a similar conch size (100 mm diameter) and the egg case also has a bag-like outline with a dark content of spherical structures^39^. The structure is 15 mm long and 14 mm wide, also larger than in the *Eleganticeras*. Since the specimen was cut and polished, the calcitic spheres inside the supposed eggs are preserved. Again, the size of the spheres is 0.5 mm in diameter, which fits well with either an initial chamber or a small ammonitella. The proportion of the entire structure is much smaller than in the *Neochetoceras*. which may be explained by larger parts still being hidden in the body chamber filling. A tomographic examination of this specimen could shed light on this question. The position of the supposed egg sac in the middle of the body chamber may be a taphonomic artifact, since normally, these reproductive organs are located more posteriorly. If one accepts the hypothesis of ammonoid ovoviviparity^42^, it could be envisioned that eggs or newly hatched ammonoids were stored in an egg case outside the ovaries.

The size ranges of the spherical structures found in the body chambers of the Triassic *Ceratites*, the Jurassic *Eleganticeras* and *Neochetoceras* are very similar and fit both with the expected initial chamber or ammonitella-diameters and with the embryo-size reported by Mironenko & Rogov^42^. The localization of these structures far behind the aperture confirms that they are likely really organs rather than other fossils trapped inside the ammonoid^63^.

#### Fecundity estimates

De Baets et al^64^. and Tajika et al^43^. estimated reproductive rates by combining ammonoid conch volumes with the volume proportions of ovaries in modern squids. Since embryo size in ammonoids has remained nearly constant since the Middle Devonian, it is reasonable to assume that egg size hardly varied thereafter. The results range between tens and tens of millions of eggs per adult female ammonoid depending on adult body size. In neocoleoids, reproductive rates lie between thirty in some octobrachians and six million in some Oegopsida^65,66^.

**Fig. 8:**
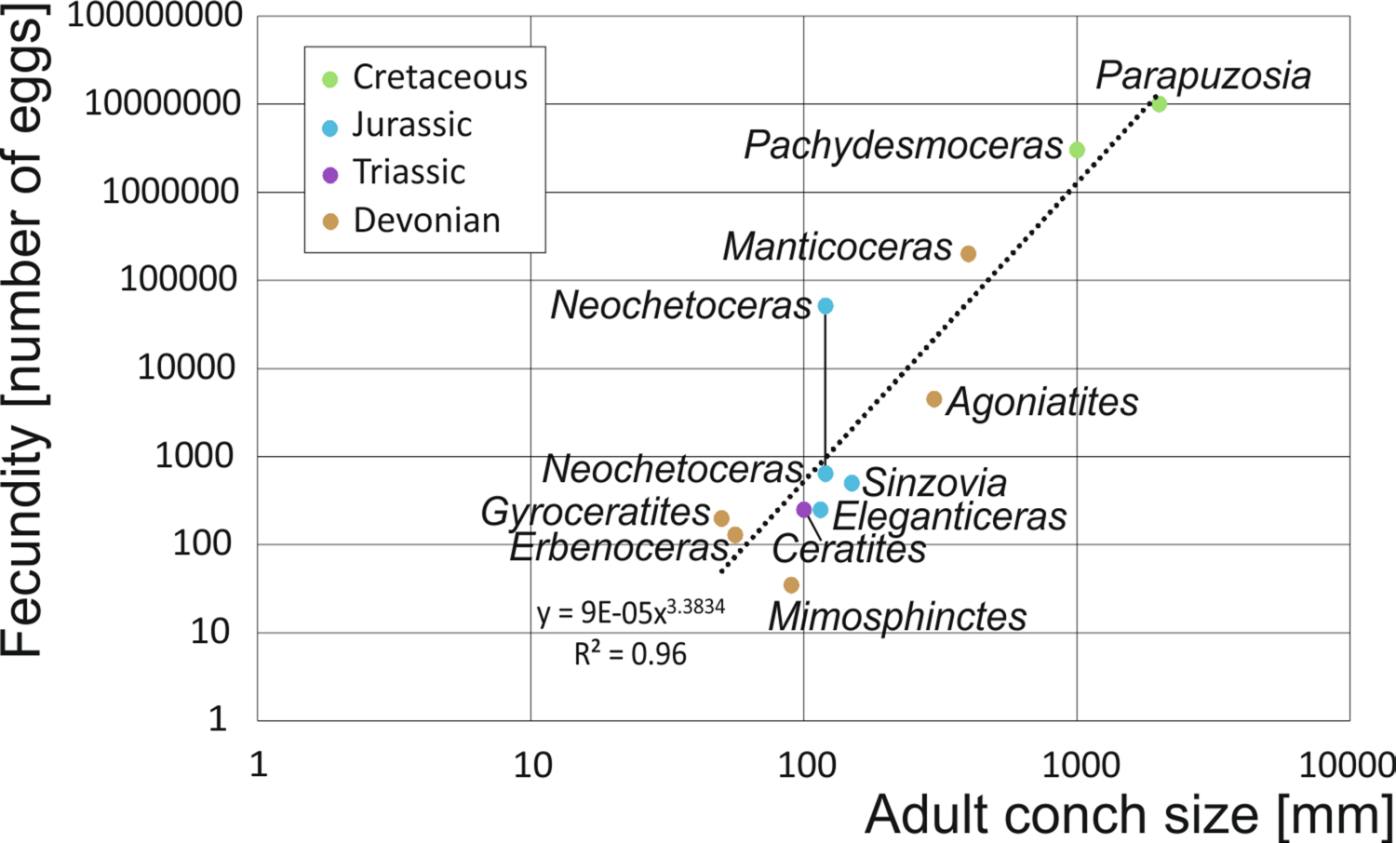
Estimates of fecundity in ammonoids, partially based on preserved eggs and on body chamber volumes (for details and references see Tb. 1).

In the new ammonite specimen, round structures interpreted as fossil remains of ovaries are well visible. With approximately 80 such structures per square centimeter, the entire 8 cm^2^ of the surface area of the organ yields an estimated total of ~ 640 such structures (Tab. 1). Assuming a similar arrangement in the third dimension and an approximate organ width of 1 cm, there could have been up to 51,200 immature eggs in the ovary of *Neochetoceras* cf. *praecursor*. This figure could have been even higher if egg size varied. In Fig. 8, we plotted these two numbers as possible limits of the fecundity range for this ammonite species. The lower estimate closely aligns with the trendline of ammonoid fecundity proposed by Tajika et al^43^., while the higher estimate exceeds it. This discrepancy can be rooted in the fact that the former authors did not consider the potential variation in egg size during development. Hence, the trendline in Fig. 8 must be interpreted as representing a conservative lower estimate.

**Table 1:** Ammonoid fecundity, published data and the new estimates included here (see Fig. 8). Conch and Egg refer to conch size and egg size; values of conch and egg sizes are given in millimeters.

Genus	Period	Conch	Fecundity	Egg	References
*Parapuzosia*	Cretaceous	2000	10,000,000	0.9	^43^
*Pachydesmoceras*	Cretaceous	1000	3,000,000	0.9	^43^
*Neochetoceras*	Jurassic	120	640	0.5	this paper, low estimate
*Neochetoceras*	Jurassic	120	51,200	0.5	this paper, high estimate
*Sinzovia*	Jurassic	50	200	1	^42^
*Eleganticeras*	Jurassic	115	250	0.5	^37^
*Ceratites*	Triassic	100	250	0.5	^39^
*Manticoceras*	Devonian	400	200,000	1.2	^64^
*Erbenoceras*	Devonian	150	500	3.7	^64^
*Mimosphinctes*	Devonian	90	35	4	^64^
*Gyroceratites*	Devonian	56	130	1.5	^64^
*Agoniatites*	Devonian	300	4500	2.3	^64^

Indirect evidence for high fecundity was provided by the high population estimates for Late Devonian ammonoid species^67^. The currently available data suggests that ammonoid fecundity correlates with conch diameter in millimeters by the third power. In turn, with the broad range of adult conch sizes in Jurassic ammonoids and the great abundance in some locality and some strata, we follow Greif et al^67^. in the conclusion that, at least in the time when young ammonoids hatched, the Jurassic seas were full of millimeter-sized ammonoids. Their immense abundance highlights the importance of ammonoid hatchlings and juveniles as food of low trophic levels of marine predators.

## Conclusions

**Fig. 9:**
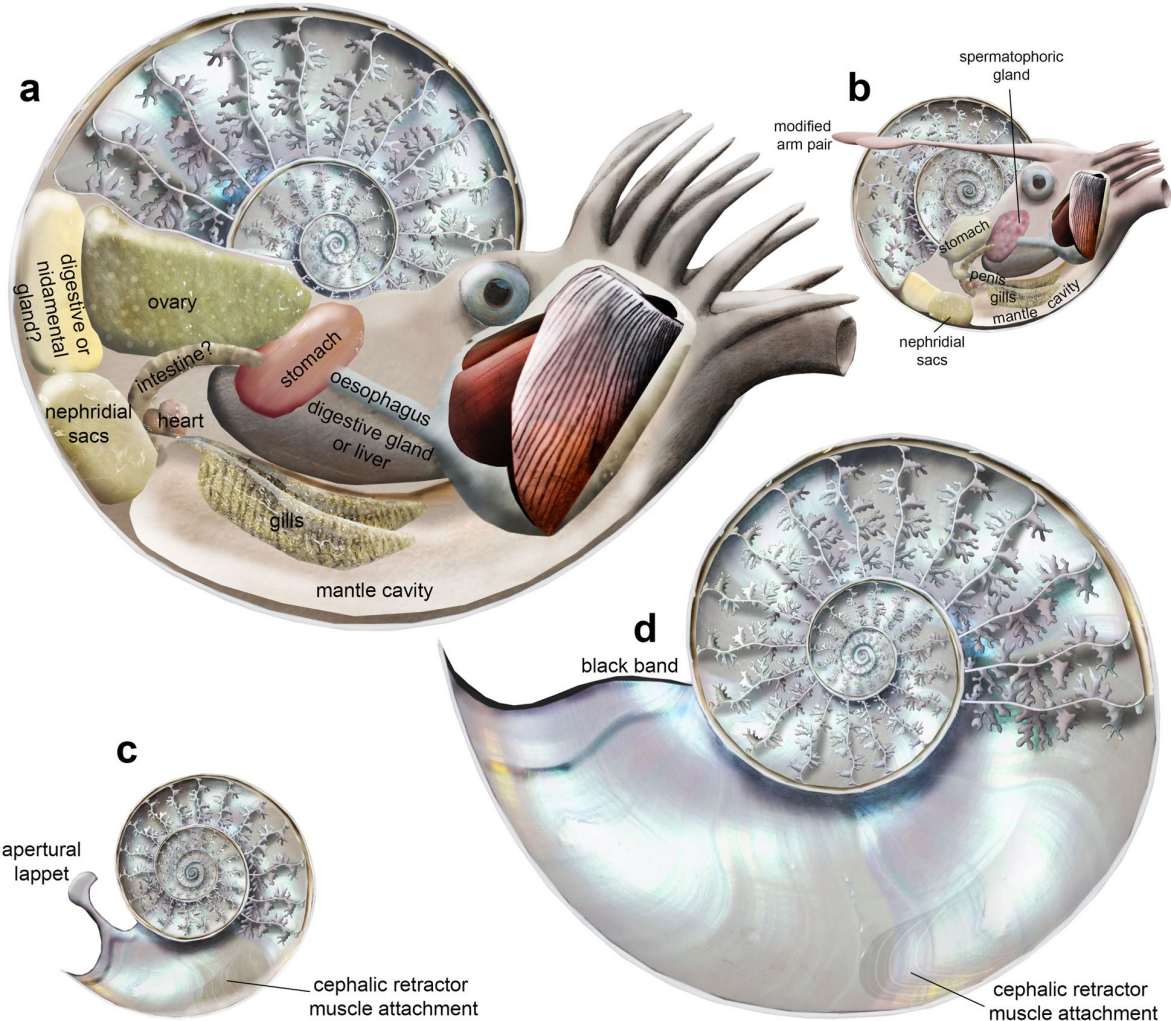
Reconstruction of the conchs and internal anatomies of males and females of the Jurassic ammonite *Neochetoceras* cf. *praecursor*. (**a**) female and (**b**) male with soft body; note that no fossilized remains of liver and funnel have been found. The male reproductive organs are reconstructed after *Subplanites*^26^. The body size proportions correspond to the adult macroconchs and microconchs^47^. Arm morphology is speculative, short arms are suggested by the faint remains in the main specimen. Insides of the empty conchs of the male (**c**) and the female (**d**), virtually cut in the plain of bilateral symmetry. The modified arm pair is reconstructed as pointing backwards because the lappet displays a posterior extension, hinting at the male approaching the female swimming backward.

The new soft tissue-ammonite from the Late Jurassic Solnhofen Archipelago is the first ammonite preserving ovaries showing egg remains in situ together associated with other organs. This further confirms the interpretation of macroconchs as females and microconchs as males (Fig. 9). Egg preservation allows estimating reproductive rates, which accordingly ranged between about 600 and over 50,000 in *Neochetoceras* cf. *praecursor*. This further confirms a rough correlation between conch diameter and fecundity in ammonoids, with a potential error of about three orders of magnitude. This error roots in the fact that this is only the third specimen preserving eggs in the ovary preserving the approximate egg size in the body chamber^37,39^. Nevertheless, these high reproductive rates are corroborated by the high abundance of ammonoids^67^, which, in turn, highlight the great ecological importance of these animals. Juvenile ammonoids were a key element near the base of trophic nets of the world oceans from their Early Devonian origin until their demise near the beginning of the Palaeogene.

## Material and methods

The new soft tissue-ammonite is permanently held in the Lauer Foundation for Paleontolgy, Science and Education (LF) (Wheaton, Illinois, USA) with the number LF 1407. It was discovered in the latest Kimmeridgian lithographic limestones (Late Jurassic strata) of the Öchselberg quarry between Breitenhill and Zandt (southern Franconia, Germany). Quite likely, the specimen comes from Beckeri Zone, Ulmense Subzone, *rebouletianum* Horizon (Torleite Formation), see Schweigert^68^ and Niebuhr & Pürner^69^. LF 1407 was originally collected and prepared by Peter Bürger. The specimen was acquired by LF in 2015.

BL and RL photographed the ammonite LF 1407 with white light (Fig. 1) and UV-light (UV wavelengths A, B, C) (Fig. 2). The use of UV light has proven to be of great benefit in visualizing phosphatized soft-tissues and their details^70,71^. Photography of the specimen was obtained with the use of a Nikon Z9 mirrorless camera using a 60 mm Nikon Macro lens. Visible light images were taken using a pair of Raleno video LED panels, set at 5600 K colour temperature, with built in diffuser and a sheet of polarizing film over the LED screen and a linear circular polarizing filter on the lens. UV light images were taken with illumination from a Way Too Cool, ‘‘triple lamp’’ equipped with appropriate filters and three 95-W bulbs which include UV A, UV B and UV C wavelengths, which were used together. An orange colour correction filter was used on the lens for UV photography as this counteracts the purple tone of the UV lights to provide clearer recognition of ultraviolet induced fluorescence (UVIF) expressed in the visible light spectrum. All images were captured using Nikon Capture 2 then rendered as focus stacked images using Heliconfocus software.

KT and AT dissected a modern *Todarodes pacificus*. This specimen had been commercially fished in the Sea of Japan (exact locality unknown), and the dead animal was purchased in a supermarket. KT and AT photographed the internal organs under normal white light for comparative anatomy.

## Data Availability

Specimen LF 1407 is permanently held with the Lauer Foundation for Paleontology, Science and Education (LF) in Wheaton, Illinois, USA. The mission of the Lauer Foundation is to curate its fossil collection to provide the scientific community and other museums with permanent access for the purposes of research, education and exhibition. Permanent access to type and figured specimens, as well as specimens listed or cited in publications together with other scientifically important specimens is guaranteed.
